# Host-Cell Type Dependent Features of Recombinant Human Aquaporin-4 Orthogonal Arrays of Particles—New Insights for Structural and Functional Studies

**DOI:** 10.3390/cells8020119

**Published:** 2019-02-02

**Authors:** Francesco Pisani, Laura Simone, Maria Grazia Mola, Manuela De Bellis, Maria Mastrapasqua, Maddalena Ruggieri, Maria Trojano, Grazia Paola Nicchia, Maria Svelto, Antonio Frigeri

**Affiliations:** 1 Department of Bioscience, Biotechnologies and Biopharmaceutic, Univ. of Bari “Aldo Moro”, 70124 Bari, Italy; francesco.pisani@uniba.it (F.P.); mariagrazia.mola@uniba.it (M.G.M.); manuela.debellis84@gmail.com (M.D.B.); graziapaola.nicchia@uniba.it (G.P.N.); maria.svelto@uniba.it (M.S.); 2 Fondazione IRCCS Casa Sollievo della Sofferenza, Cancer Stem Cells Unit, 71013 San Giovanni Rotondo (FG), Italy; lasimone@gmail.com; 3 School of Medicine, Basic Medical Sciences, Neuroscience and Sense Organs, Univ. of Bari “Aldo Moro”, 70124 Bari, Italy; marymastra@libero.it (M.M.); maddalena.ruggieri@uniba.it (M.R.); maria.trojano@uniba.it (M.T.); 4 Institute of Biomembranes, Bioenergetics and Molecular Biotechnologies, National Research Council, 70126 Bari, Italy

**Keywords:** aquaporin-4, OAPs isolation, OAPs structure, neuromyelitis optica

## Abstract

The CNS plasma-membrane water channel aquaporin-4 (AQP4) is expressed as two major isoforms able to aggregate into supramolecular assemblies known as ‘orthogonal arrays of particles’ (OAPs). OAP subnanometric features are largely unknown mainly because a method for the expression, isolation, and crystallization of integral human OAPs has not been developed. Here, the human OAP-forming isoform M23-AQP4 was expressed in insect and mammalian cell lines and AQP4 and OAP features evaluated. Native size exclusion chromatography was employed to isolate and analyze authentically folded OAPs, and neuromyelitis optica (NMO)-specific sandwich ELISA was developed to test OAP-integrity. The results demonstrate that in insect cells most AQP4 remains intracellular and unfolded and that OAPs are largely disassembled after the detergent extraction step. In mammalian cells, AQP4 showed regular plasma membrane targeting and OAPs exhibited strong post-extraction stability. Starting from the mammalian cell expression system, we isolated authentically folded OAPs. Together these data suggest a new strategy for expressing and isolating integral recombinant human OAPs and providing new insights into the cell-type dependent OAP-assembly and post-extraction stability, potentially useful to design new approaches for structural and functional studies of OAP and for other plasma membrane proteins organized into supramolecular structures.

## 1. Introduction

Aquaporin-4 (AQP4) is the main astrocytic cell-membrane water channel of the central nervous system (CNS) where it contributes to the maintenance of water-ion homeostasis, to the functionality of the blood-brain-barrier and seems to play a role in the glymphatic pathway [1,2,3,4]. AQP4 is expressed as two main isoforms M1-AQP4 (32KDa) and M23-AQP4 (30KDa), while other isoforms have also been described recently [5,6,7]. AQP4 monomers, similar to those of other aquaporins are organized in tetramers, but AQP4 heterotetramers are specifically able to further aggregate in supramolecular assemblies, known as orthogonal arrays of particles (OAPs). OAPs are well-ordered plasma membrane structures visible by freeze fracture electron microscopy as two-dimensional arrays [8,9]. OAP organization confers specific AQP4 localization [10,11,12,13], tissue-distribution [14,15] and is involved in some pathological processes [16,17,18,19]. The physiological role of OAPs is unclear and little is known about the molecular basis of OAP assembly and stability. Biophysical and biochemical evidence shows that M1-AQP4 destabilizes while M23-AQP4 stabilizes OAP assembly [12,20,21] through M23-specific N-terminal residue interaction [12,20]. OAP assembly appears to be a plasma membrane process that involves not yet well-identified cell-membrane-specific factors [22]. Although OAPs have been visualized and analyzed at nanometrical resolution with super-resolution microscopy approaches [6,10,23,24], sub-nanometrical resolution is required to deeply investigate the molecular properties of OAPs. The crystallographic technique reaches this resolution but, to date, available AQP4 crystals are only tetramers (PDB IDs: 3GD8; 2ZZ9; 2D57), while OAP crystals are not available and consequently the molecular organization of OAPs remains unknown. This represents one of the main obstacles for the development of a new functional hypothesis based on the knowledge of OAPs’ molecular properties. Although the monomeric-related functions, such as water conductance and the monomer-to-monomer assembly into the tetramer, have been well established using X-ray crystal data, followed by dynamic simulation and wet functional experiments [25,26,27], OAP properties could be only partially explained starting from tetrameric crystal data. In this respect, previous data indicate that the routine methods for AQP4 expression and purification used for AQP4 crystal production are poorly compatible with OAP integrity [23]. Therefore, the identification of an appropriate OAP expression system and the development of an enrichment/purification methods of OAPs in native form are needed to isolate authentically folded OAPs to perform OAP structural and functional studies. Moreover, the isolation of authentically folded OAPs could support the development of molecular tools for the diagnosis of AQP4-related diseases such as neuromyelitis optica (NMO) and could represent a useful experimental protocol for the study of other plasma membrane proteins organized into supramolecular assembly state. For instance, to date the detection of AQP4-IgG autoantibodies (NMO serum diagnostic marker) is performed by a time-consuming cell-based assay requiring a skilled operator, while an easy method based on isolated human OAPs as molecular target is not available [18,19,28,29,30,31,32,33].

Considering that unknown factor(s) may be involved in the natural OAPs assembly and stability, reproducing these conditions in a recombinant expression system represents a challenge, especially for those regarding the post-extraction stability of OAPs, as already demonstrated for other proteins [34].

Here we tested the possibility of producing and isolating authentically folded recombinant human OAPs in insect and mammalian cell lines. The AQP4 expression levels, cellular localization and folding, OAP assembly, and post-extraction stability were analysed.

We provide new insights into the species- and cell-type dependent OAP assembly and post-extraction stability and lay the foundation for the isolation of authentically folded OAPs for structural and functional studies and for the development of other applications for which OAPs-assembly is mandatory.

## 2. Materials and Methods

### 2.1. Cell Line

The DI TNC1 cell line, established from primary cultures of type 1 astrocytes from brain diencephalon tissue of one-day-old rats (www.lgcstandards-atcc.org) was grown in DMEM medium supplemented with 10% heat-inactivated foetal bovine serum (FBS), 100 UI/mL penicillin, and 100 mg/mL streptomycin, and maintained at 37 °C in a 5% CO_2_ incubator.

The Sf9 cell line derived from the pupal ovarian tissue of the fall army worm, *Spodoptera frugiperda*, from the parental IPLBSF-21 (Sf21) cell line (www.thermofisher.com), was grown in Supplemented Grace’s Insect Medium with 5% heat-inactivated foetal bovine serum (FBS), 100 UI/mL penicillin and 100 mg/mL streptomycin, in adherent culture and maintained at 27 °C without CO_2_ exchange. Suspension cultures were prepared in serum-free Sf-900™ II SFM in a shaker apparatus (Thermo Fisher Scientific, Waltham, MA, USA) shaking at 130 rpm in a non-humidified incubator at 26–28 °C.

The Free Style 293-F cell line (HEK-FS), derived from the 293 parental cell line established from primary embryonic human kidney (www.thermofisher.com), was grown in Free Style 293 Expression Medium in a sterile Erlenmeyer flask and maintained at 37 °C in an incubator (Thermo Fisher Scientific, Waltham, MA, USA) containing a humidified atmosphere of 8% CO_2_ in air on an orbital shaker platform rotating at 135 rpm. Cell culture reagents were purchased from Thermofisher (Thermo Fisher Scientific, Waltham, MA, USA, www.thermofisher.com).

### 2.2. Transfection and Baculovirus-Based Gene Expression

Human AQP4-M23 CDS was cloned into pTarget (www.promega.com) and used for the transfection procedure. To obtain stable expressing DI TNC1-AQP4-M23 cell lines, 24 h before transfection, the cells at 70% confluence were plated using antibiotic-free medium cells. Transfection was carried out with Lipofectamine 2000 Reagent mix following the manufacturer’s instructions (www.thermofisher.com) and stable clones were selected after 36 h by G418 treatment.

On the day of transfection, HEK-FS cells were resuspended to obtain a concentration of 1.1 × 10^6^. cells/mL and the cells were checked for extensive clumping and viability. Transient transfection was carried out using 293Fectin in OptiMEM growth medium (www.thermofisher.com) according to the manufacturer’s protocol and analysed after 36 h. Human AQP4-M23 CDS was expressed in Sf9 insect cells using the Bac-to-Bac expression system (www.thermofisher.com). A bacterial strain (DH10Bac) was transformed with M23-recombinant plasmid and colonies containing recombinant bacmid were grown for isolation of the resulting DNA according to the manufacturer’s instructions (www.thermofisher.com). This recombinant bacmid was used to transfect Sf9 cells in monolayer. Supernatants of transfected Sf9 cells were used to infect more Sf9 cells through two stages of amplification. Sf9 cells (1 × 10^6^/mL) were then infected with recombinant viruses using 1, 5, and 10 multiplicity of infection (MOI) and infected cells were harvested 72h after infection. Purified AQP4-M23-6xHis from Sf9 cells [23] was purified by nickel based chromatography using Agarose-Ni^2+^ NTA beads (www.qiagen.com) according to the instruction manual by batch purification, checked by SDS-PAGE and Coomassie staining, quantified using the Micro-BCA Protein Assay Kit (Thermo Fisher Scientific, Waltham, MA, USA, www.thermofisher.com). All constructs were fully sequenced (www.agilent.com).

### 2.3. Antibodies

The following primary antibodies were used: goat polyclonal anti-AQP4 (C-19) (sc-9888, 1:500) rabbit polyclonal anti-AQP4 AQP4 (H-80) (sc-20812), goat polyclonal anti-calnexin, (sc6465, 1:250) (Santa Cruz Biotechnology, Dallas, TX, USA); rabbit polyclonal anti-actin (A42066, 1:500) (www.sigmaaldrich.com); goat anti-human IgG antibody, biotin conjugate (AP112B, 1:7000) (www.merckmillipore.com).

The secondary antibodies used for immunofluorescence analysis were: donkey anti-goat Alexa Fluor 488-conjugated (A11055) and goat anti-human Alexa Fluor 488-conjugated (A11013) (www.thermofisher.com).

The following secondary antibodies were used for Western blot: donkey anti-goat IgG-HRP (sc-2020) and goat anti-rabbit IgG-HRP (sc-2004).

The secondary antibody used for ELISA was streptavidin, peroxidase conjugated (SA202, 1:1000) (www.merckmillipore.com).

### 2.4. Patient Sera

58 sera were used, 34 sera from NMO patients [35], and 24 from controls. Controls included five patients with definite Multiple Sclerosis (MS), five myasthenic, four with a recurrent form of myelitis, two with polyneuropathy, one with myopathy, and seven healthy donors. The diagnosis of MS was established according to the McDonald criteria [36].

All subjects gave their written informed consent to the study, which was approved by the Institutional Ethics Committee of the University of Bari.

### 2.5. Immunofluorescence

#### 2.5.1. AQP4 Immunofluorescence

After expression, Sf9 and HEK-FS cells were seeded onto a poly-L-lysine-coated coverslip and allowed to attach for 3 h. Sf9, HEK-FS, and DI TNC1 cells were fixed in 4% paraformaldehyde for 15 min, washed three times in phosphate-buffered saline (PBS), permeabilized with 0.3% Triton X-100 in PBS, blocked using 2% bovine serum albumin (BSA) diluted in PBS for 30 min at room temperature (RT), incubated for 1 h with C-terminal specific commercial anti-AQP4 primary antibody, washed with PBS-BSA, incubated with Alexa Fluor-conjugated secondary antibody and mounted with a mowiol mounting medium.

#### 2.5.2. AQP4-IgG Immunofluorescence

AQP4-IgGs binding was performed using live Sf9, HEK-FS and DI TNC1 cells: cells were blocked using PBS-2%BSA for 30 min, incubated with NMO serum at RT for 1 h, washed with PBS-BSA, incubated with Alexa Fluor-conjugated secondary antibody, fixed for 10 min with 4% formaldehyde in PBS and mounted with the mounting medium.

Immunostained cells were observed with a photomicroscope equipped for epifluorescence and 16×, 40× oil PL FL FLUOTAR objectives (Wetzlar, Germany, www.leica-microsystems.com). Digital images were obtained with a DMX1200 camera (Wetzlar, Germany, www.leica-microsystems.com) and processed using LAS AF software (version 3.2.0, www.leica-microsystems.com). Once captured, the auto contrast function was applied to the whole images using Photoshop CS5 (San Jose, CA, USA).

### 2.6. Confocal Imaging and Analysis

Confocal images were acquired with an automated inverted Leica TCS SP2 AOBS system confocal microscope equipped with an HCX PL APO ×63/1.4NA oil-immersion objective (www.leica-microsystems.com). All confocal images were collected using a 488 nm laser line for excitation and a pinhole diameter of 1 Airy unit.

### 2.7. DDM/SDS Solubility Assay and Western Blotting

The DDM/SDS solubility assay was performed as already described [34] with some modifications. Briefly, after expression, cells were washed twice in phosphate-buffered saline (PBS), pelleted (1200× *g* for 5 min) and resuspended at 1 × 10^6^/mL in ice-cold cell buffer (50 mM Tris (pH 7.4) and 150 mM NaCl) added with a cocktail of protease inhibitors (www.merckmillipore.com).

Cell suspensions were sonicated briefly and the total protein concentration was measured with a bicinchoninic acid (BCA) Protein Assay Kit (www.thermofisher.com). Sample were then solubilized in the indicated detergent (SDS and DDM) at 1% (*w*/*v*) final concentration at either 4 °C for DDM or 20 °C for SDS for 1 h. The lysate was centrifuged at 17,000× *g* for 30 min at 4 °C to remove the insoluble fraction.

Equal amounts relative to the cell lysates (10 μg total protein/lane) were dissolved in Laemmli Sample Buffer (www.bio-rad.com) and 50 mM dithiothreitol, heated to 37 °C for 10 min, loaded and separated by SDS-PAGE on a 13% polyacrylamide and transferred to polyvinylidene difluoride (PVDF) membranes (www.merckmillipore.com).

After transfer, the membranes containing the blotted proteins were blocked and incubated with primary antibodies diluted as described in the Antibodies section (Section 2.3). After washing, the membranes were incubated with peroxidase-conjugated secondary antibodies and washed again. Reactive proteins were revealed with an enhanced chemiluminescent detection system (ECL-Plus, www.thermofisher.com) and visualized on a ChemiDoc imaging system (www.biorad.com). The measure of “DDM-solubilized” was obtained as the ratio of the DDM and SDS signals (DDM/SDS (%)).

### 2.8. Blue Native-PAGE and 2DE

Sf9, HEK-FS, and DI TNC1cells were washed twice in PBS, pelleted (1200× *g* for 5 min) and dissolved in seven volumes of BN buffer (1% DDM, 12 mM NaCl, 500 mM 6-aminohexanoic acid, 20 mM Bis-Tris, pH 7.0, 2 mM EDTA, 10% glycerol) plus Protease Inhibitor Cocktail as previously reported [37]. The cells were lysed on ice for 1 h, and the samples were then centrifuged at 17,000× *g* for 30 min at 4 °C. The supernatants were collected, and the total protein content was calculated using the BCA Protein Assay Kit (Thermo Fisher Scientific, Waltham, MA, USA). Twenty micrograms of protein sample were mixed with 5% CBB G-250 (Coomassie blue G-250) and loaded onto a polyacrylamide native gradient gel (3–9%) [38]. The running buffers were as follows: anode buffer (25 mM imidazole, pH 7) and blue cathode buffer (50 mM tricine; 7.5 mM imidazole; 0.02% Coomassie blue G-250; pH 7). For the 2D BN/SDS-PAGE analysis, lanes from the first dimension were cut into individual strips and equilibrated in denaturing buffer (1% SDS and 1% β-mercaptoethanol) for 1 h at RT and placed in a 2D SDS-PAGE with the same thickness. Separation of the second dimension was performed in a 13% SDS-polyacrylamide gel at 25 mA per gel. At the end of the run, the gel was blotted onto a PVDF membrane for Western blot analysis.

### 2.9. Preparation of Membrane Vesicles

Membrane vesicles from DI TNC1-AQP4 and Sf9-AQP4 expressing cells were prepared as previously described with minor modifications [39]. Cells from 10 150 m diameter plastic dishes for DI TNC1 and 500 mL of cell cultures for Sf9 were harvested, washed two times with Ca^2+^/Mg^2+^-free PBS, scraped in homogenizing buffer (HB, 300 mM sucrose, 1 mM EDTA, 10 mM Tris–HCl, pH 7.2), added with a protease inhibitor cocktail and homogenized by five strokes with a Potter-Elvehjem homogenizer. The homogenate was spun at 4000 *g* for 15 min, and the supernatant was centrifuged at 17,000 g for 45 min to obtain a fraction enriched in plasma membranes.

### 2.10. Native Size Exclusion Chromatography

Proteins from the plasma membrane-enriched fraction were extracted on ice for 1 h, vortexed every 5 min in 7 volumes of Extraction Buffer (500 mM aminocaproic acid, 50 mM imidazole, 2 mM ethylenediaminetetracetic acid (EDTA), 3% n-Dodecyl **β**-D-maltoside (DDM) and a protease inhibitor cocktail) was added with 12 mM or 150 mM NaCl. Then it was centrifuged at 22,000× *g* for 30 min at 4 °C, and the supernatant was used for ELISA and nSEC experiments.

Briefly, lysate was injected into AKTA-FPLC using the Sephacryl S-500 stationary phase, high prep 16/60 (www.gehealthcare.com). All chromatographic phases were performed at RT, max 0.15 MPa of column pressure, and 1 mL/min of flux rate. Columns were first equilibrated with two column volumes of nSEC-buffer-0.15% DDM (500 mM aminocaproic acid, 50 mM imidazole, 2 mM EDTA, 0.15% DDM, 150 mM NaCl), and then injected with 5 mL of protein lysate (10 mg/mL) in 16/60 columns. The absorbance at 280 nm was continuously monitored and fractions of 1 mL for the 16/60 column. Total protein content of nSEC fractions was quantified using a Micro-BCA Protein Assay Kit (www.thermofisher.com).

### 2.11. Dot Blot

After the nSEC, the AQP4 elution profile was evaluated by dot blot. Two microlitres of each fraction was spotted onto a nitrocellulose membrane (www.merckmillipore.com) blocked with 5% milk in 1% Triton X-100 in PBS and processed as reported for regular AQP4 immunoblotting.

### 2.12. Sandwich ELISA

The ELISA was performed as previously described [40]. Briefly, to detect NMO binding Maxisorp NUNC Plates (www.thermofisher.com) were coated with 0.2 μg of commercial goat anti-AQP4 antibody (Santa Cruz, Santa Cruz Biotechnology, (www.scbt.com) overnight at 4 °C or for 2 h at 37 °C. After coating, wells were washed and coated with approximately 3, 7, and 15 μg of total protein per well from the vesicle extract. Negative control wells were only coated with the buffer. After incubation for 1 h, sera diluted from 1:1000 to 1:16,000 and anti-AQP4 antibody were incubated in the wells for 1 h under shaking. Wells were then washed, incubated with anti-human biotinylated secondary antibody (see Section 2.3), washed again and incubated with streptavidin-HRP (see antibodies section). After 1 h, 100 μL of TMB solution was added (www.merckmillipore.com) for 20 min and the reaction was stopped by adding 100 μL of 0.3 M sulphuric acid solution. Finally, absorbance was read at 450 nm using the Flex Station 3 plate reader (www.moleculardevises.com). The NMO normalized absorbance was calculated as follows: absorbance of AQP4-coated well minus absorbance of negative control well.

To detect the amount of AQP4 coating the wells, OAP-ELISA was performed using 0.2 μg/well of commercial rabbit anti-AQP4 (www.scbt.com) in the first coating step. Goat anti-AQP4 antibody (www.scbt.com) and the anti-goat Biotin (Millipore, Burlington, MA, USA, www.merckmillipore.com) were used.

AQP4-IgG binding was normalized to the amount of AQP4 as the ratio of ELISA signals (AQP4-IgG/AQP4 (%)). This percentage indicates the AQP4-IgG affinity to the AQP4. The analyses were performed in a blind manner by three independent investigators.

The E14 and E30 fractions enriched in AQP4 of nSEC analysis from DI TNC1 and Sf9 vesicles, respectively, were used to perform ELISA. Sensitivity and selectivity were calculated as described [40].

### 2.13. Densitometric Analysis

Densitometric analysis was performed using ImageJ software (version 2.0.0). Optical density value was determined for equal sized boxes drawn around antibody-stained bands (www.imagej.nih.gov) and analyzed by GraphPad Prism 6 (www.graphpad.com/scientific-software/prism).

### 2.14. Statistical Analysis

Statistically significant differences were computed using the Student’s *t*-test and two-way ANOVA analysis, the level being set at *p* < 0.05. All statistical analysis was performed using GraphPad Prism version 6 (www.graphpad.com/scientific-software/prism). Data are expressed as mean ± SEM. The experiments were repeated from three to five times as indicated in the figure legends

## 3. Results

### 3.1. Analysis of AQP4 Localization and OAP Assembly into the Cell Membrane in Insect and Mammalian Cell Lines

**Figure 1:**
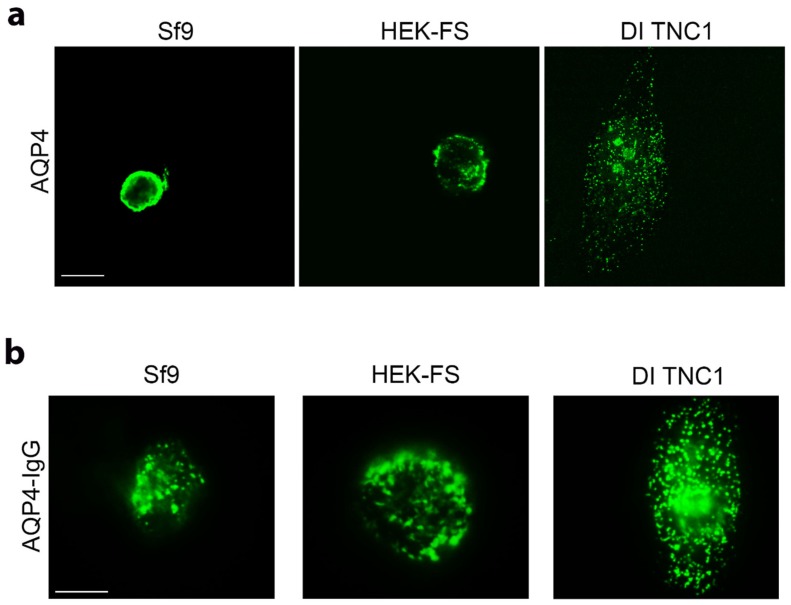
Analysis of AQP4 localization and OAP assembly in the cell membrane in insect and mammalian cell lines. (**a**) Confocal single scan images of AQP4 expressing Sf9, HEK-FS, and DI TNC1 cells stained with anti-AQP4 antibody (green). Scale bars 20 μm. (**b**) Representative epifluorescence images of immunofluorescence performed with AQP4-IgGs containing NMO serum, in green, on Sf9, HEK-FS, and DI TNC1. Scale bars 10μm.

The OAP-forming human M23-AQP4 was expressed in Sf9 insect cells, in human embryonic kidney HEK-FS cells and in astrocyte DI TNC1 cells. Sf9 and HEK-FS are widely used for large-scale protein production, while DI TNC1s are rat astrocyte-derived cells. AQP4 expression and localization was evaluated by immunofluorescence (IF) with anti-AQP4 antibody. Confocal analysis of stack section showed a weak intracellular staining in mammalian cells compared to that observed in insect cells (Figure 1a). In particular, Sf9 cells appear to include a circular fluorescence within the cytosol immediately below the membrane level. This may be due to the swelling of the nucleus and other compartments such as ER that occurs in insect cells following baculovirus infection. DI TNC1 shows typical plasma membrane punctate staining while intracellular staining is not appreciable also because DI TNC1s are very thin cells (3–4 μm in thickness).

To assess whether AQP4 is correctly assembled into OAPs at the plasma membrane level, immunofluorescence experiments and widefield fluorescence analysis using AQP4-IgG containing NMO serum as primary antibody, which specifically recognizes extracellular epitopes of OAPs, were performed. Extracellular AQP4-IgG binding was observed in all cell lines (Figure 1b), suggesting that AQP4 is correctly assembled in OAPs in the cell membrane.

### 3.2. Analysis of AQP4 Folding in Insect and Mammalian Cell Lines

**Figure 2:**
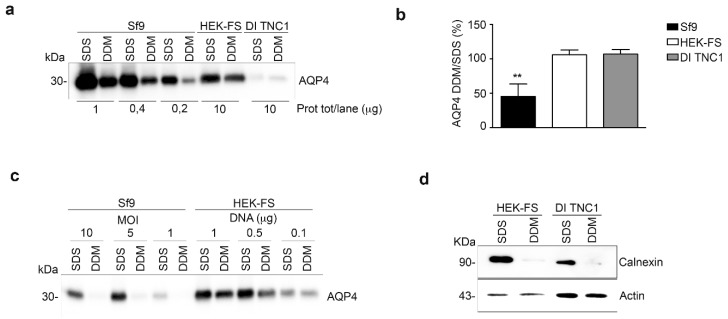
Analysis of AQP4 folding in insect and mammalian cell lines. (**a**) Western blot of AQP4 solubilized from whole insect (Sf9) and mammalian cells (HEK-FS and DI TNC1) using two different detergents (SDS and DDM) and probed with an anti-AQP4 antibody. Three different amounts of total protein are shown for the Sf9 cell line, while a higher amount of total protein is shown for mammalian cells, as indicated. (**b**) Histochart of Western blot data reported in a showing the percentage of DDM-solubilized AQP4 (DDM/SDS (%)), determined by comparing the amount of AQP4 in the DDM-solubilized fraction to the amount of AQP4 in the SDS-solubilized fraction. Data are plotted as mean ± SEM (** *p* < 0.005, *n* = 3). (**c**) Immunoblot detection of AQP4 content in SDS and DDM-solubilized fraction from insect and mammalian cells infected/transfected using different infection/transfection condition as indicated in each lane. (**d**) Immunoblot detection of Calnexin containing SDS and DDM-solubilized fraction from whole HEK-FS and DI TNC-1 cells. Actin was used to normalize for equal loading.

As recently reported [34], the ratio between DDM and SDS solubility of a plasma membrane protein expressed in heterologous systems is an indicator of its correct folding. Sf9, HEK-FS, and DI TNC1 expressing M23-AQP4 were lysed, proteins extracted by DDM or SDS and the AQP4 content in the soluble fractions measured by Western blotting (Figure 2a). As expected, the baculovirus system was able to express considerably more total AQP4 protein than the mammalian expression systems (AQP4 SDS-signal vs. total protein/lane in Figure 2a). In mammalian cells (HEK-FS and DI TNC1); however, the ratio of DDM/SDS signals of AQP4 was found to be the highest and at least twice that measured in insect cells (Sf9) (Figure 2b). To test the possibility that the low DDM/SDS ratio of M23-AQP4 observed in Sf9 cells could be due to the very high expression levels of protein in Sf9 cells, the infection was performed by decreasing the MOI (multiplicity of infection) for Sf9 and reducing the DNA amount for HEK-FS cells. Western blot analysis showed that the DDM/SDS ratio appeared to be unaffected by MOI in insect cells or by the DNA amount in HEK-FS cells (Figure 2c). To evaluate whether the DDM is able to extract proteins also from intracellular membrane compartments and in particular from the endoplasmic reticulum (ER), the level of the ER-marker Calnexin was measured by Western blot in the SDS- and DDM-solubilized fractions. The results show that DDM was unable to solubilize the ER compartment in either HEK-FS or DI TNC1 (Figure 2d).

### 3.3. Analysis of OAPs Post-Extraction Integrity Performed by Blue-Native SDS/PAGE and Native Size Exclusion Chromatography (nSEC)

**Figure 3:**
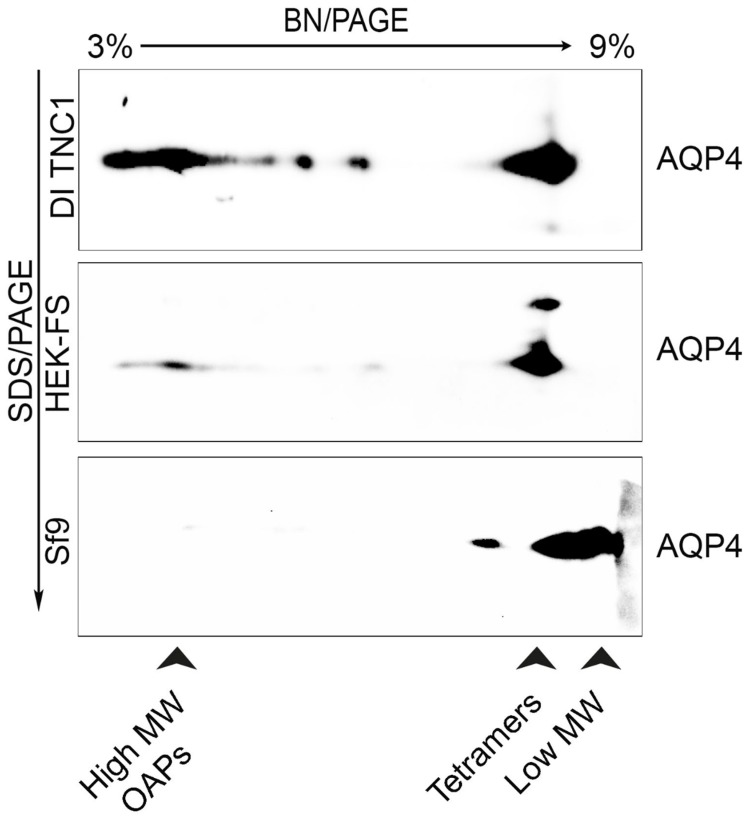
Analysis of OAPs post-extraction integrity performed by 3–9% BN-SDS/PAGE (2DE) in the DDM-solubilized fraction.

**Figure 4:**
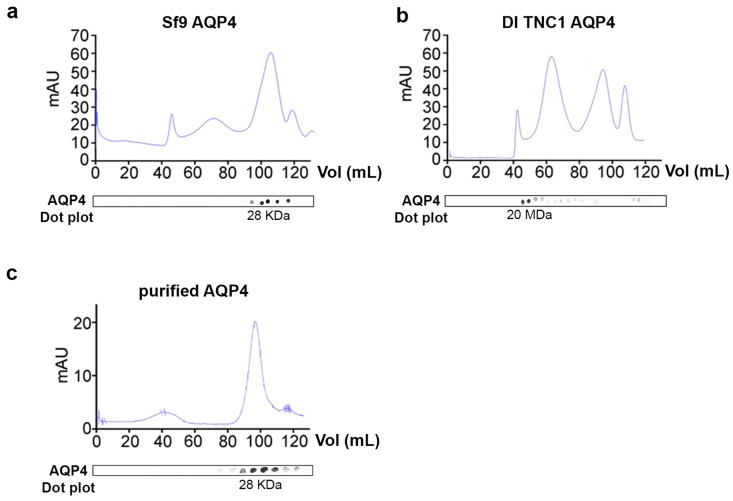
Analysis of OAPs post-extraction integrity performed by native size exclusion chromatography (nSEC). (**a**) Chromatogram obtained by nSEC for vesicles from Sf9-M23-AQP4 expressing cells. (**b**) Elution profile for vesicles derived from DI TNC1-M23-AQP4 expressing cells. (**c**) Elution profile for HIS-tagged and nickel-purified M23-AQP4 expressed in Sf9 cells. AQP4 levels in each fraction were evaluated by dot blot displayed at the bottom of each chromatogram.

To evaluate supramolecular features of recombinant AQP4 and the post-extraction integrity of OAPs, DI TNC1-, HEK-FS-, and Sf9-M23-AQP4 expressing cells were extracted by DDM in native conditions the soluble fraction was analyzed by 13% SDS-PAGE followed by 3–9% gradient BN-SDS/PAGE (2DE) and by AQP4 immunoblotting. 2DE analysis showed that high molecular weight OAPs were present in the total extract obtained from DI TNC1 and were less abundant in HEK-FS extract. Notably, Sf9 total extract contained mainly tetramers and the lower molecular weight (MW) AQP4 pool (Figure 3). To exclude the possibility that total protein extract from Sf9 cells contained mainly an AQP4 pool deriving from ER where AQP4 resides in tetrameric form [22], plasma membrane-enriched fractions were prepared from Sf9- and DI TNC1-M23-AQP4 expressing cells as already reported [22]. Vesicle extracts obtained were subjected to high-resolution size exclusion chromatography (nSEC) and the AQP4 level in each eluted fraction was estimated by dot blot analysis (Figure 4). Sf9 AQP4 was eluted in a later fraction interval (elution volume of 100 mL) (Figure 4a) compared to DI TNC vesicle extract (elution volume of 40 mL) (Figure 4b) suggesting different OAP-integrity profiles in the cell lines. Interestingly, the elution volume of AQP4 expressed in Sf9 was similar to that observed for purified AQP4 expressed in the same cells (Figure 4c). The results show that the DDM-solubilized fraction from DI TNC1 contains integral post-extraction OAPs, while OAPs are largely disassembled in Sf9 extract. These data, in agreement with those obtained with 2DE, confirm the different plasma membrane behaviour of AQP4 in mammalian compared to transfected insect cells.

Thus, despite Sf9 cells also being able to assemble OAPs in the cell membrane as suggested by AQP4-IgG based IF (Figure 1b), the post-extraction integrity of OAPs was found to be higher in mammalian than in insect cells.

Immunoblot of AQP4 in DI TNC1, HEK-FS, and Sf9 cell lines after 13% SDS-PAGE and 3–9% gradient BN-PAGE. The arrowheads indicate AQP4 pool visible in the protein extracts. High MW OAPs are only visible in mammalian cells, especially in DI TNC1.

### 3.4. Evaluation of OAP Post-Extraction Stability by AQP4-IgGs Binding in nSEC Elutes and Plasma Membrane Vesicles Extracts

**Figure 5:**
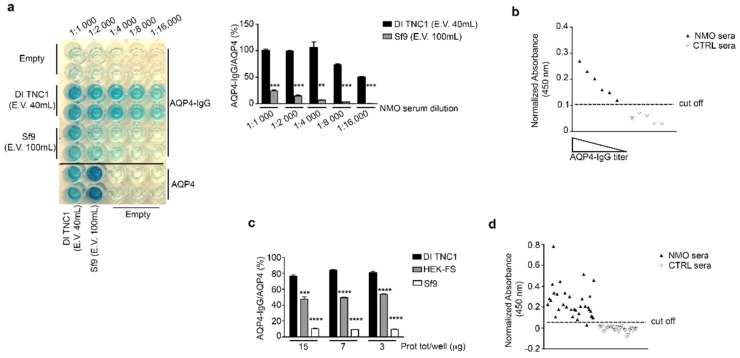
Analysis of the AQP4-IgG binding by sandwich ELISA using nSEC elutes and cell-membrane vesicle extract. (**a**) AQP4-IgG ELISA using nSEC elute E.V.40 mL from DI TNC1 and E.V.100 mL from Sf9. Note that the AQP4-IgG binding was strongly evident up to 16,000 fold time dilution of NMO sera only for DI TNC1 elute. Data are plotted as mean ± SEM (*** *p* < 0.0005 ** *p* < 0.005, *n* = 3). (**b**) Testing E.V.40 mL from DI TNC1 nSEC for AQP4-IgG binding using six different NMO sera and five different control sera. The AQP4-IgG binding (normalized absorbance at 450 nm) was found to be specific for NMO population and proportional to the AQP4-IgG titer. (**c**) AQP4-IgG ELISA using Sf9, DI TNC1, and HEK-cell-membrane vesicles extracted by DDM. Note that the AQP4-IgG binding was extremely low for Sf9, and maximal for DI TNC1. Data are plotted as mean ± SEM (** *p* < 0.005, *** *p* < 0.0005, **** *p* < 0.0001, *n* = 3). (**d**) Measure of sensitivity and selectivity of sandwich AQP4-IgG ELISA using HEK-FS cell-membrane vesicles. Sensitivity of 97% (33/34) and a selectivity of 100% (24/24) were obtained.

The E.V.40 mL (elution volume of 40 mL) and E.V.100 mL (elution volume of 100 mL) coming from nSEC separation of DI TNC1 and Sf9 vesicles, respectively, were tested for the binding to AQP4-IgG-containing NMO serum in ELISA sandwich. The results show that the binding affinity (AQP4-IgG/AQP4 (%)) using the Sf9 E.V.100 was at least five-fold lower than that obtained using the E.V.40 from DI TNC1 cells (Figure 5a). Next, the E.V.40 of DI TNC1 nSEC was tested on a small population of control and NMO sera. Data show that the normalized absorbance of the E.V.40 of DI TNC1 was specific and proportional to AQP4-IgG titer, confirming that E.V. 40 mL of DI TNC1 contains integral OAPs (Figure 5b).

To exclude the possibility that the absence of OAPs in elute from Sf9 vesicles separation was due to hydrostatic and kinetics forces typical of the chromatographic approach, the plasma membrane vesicles extracted by DDM without any further enrichment were used as target in AQP4-IgG sandwich ELISA assay. Once again, the percentage of AQP4-IgG/AQP4 binding was highest for DI TNC1, intermediate for HEK-FS and lowest for Sf9 vesicles (Figure 5c).

**Figure 6:**
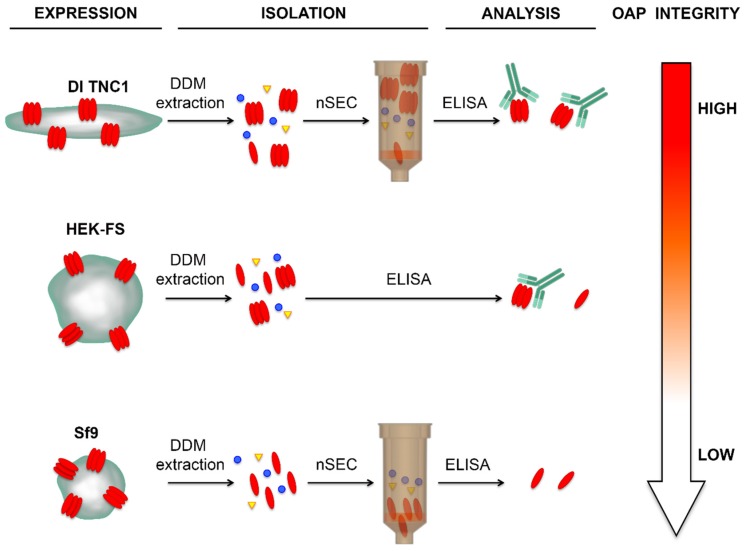
Schematic diagram of the expression, isolation, and integrity of OAP in different cell lines.

Despite the highest AQP4-IgG affinity being observed for DI TNC1, to perform a large-scale ELISA assay potentially useful for NMO diagnostic purposes it is necessary to use HEK-FS due to the higher AQP4 yield. Plasma membrane vesicle extract from HEK-FS was used as target in ELISA screening of a large population of NMO and control sera. The sensitivity and selectivity obtained were 97% (33/34) and 100% (24/24), respectively (Figure 5d). The same sera population was tested using our home-made CBA test [28], obtaining the same results (data not shown). These data confirm the integrity of OAPs in the HEK-FS extract. Figure 6 shows the summary of results here obtained.

All three host-cells were able to express recombinant AQP4 organized into OAPs at the plasma membrane level. The DDM extraction step gave high OAPs-post extraction stability in astrocyte-derived DI TNC1 cells compared to HEK-FS cells, while in Sf9 AQP4 was extracted in tetrameric form. The nSEC enrichment method was found to be compatible with the isolation of authentically folded OAPs as indicated by AQP4-IgG binding.

## 4. Discussion

The isolation of integral plasma membrane proteins in the supramolecular assembly state remains a big challenge and plays a key role in studying the structure and the functional role of these proteins. Indeed, as a result of the PDB database (PDB, www.rcsb.org) search, of a total of 216 crystals of membrane proteins only two are referred to proteins organized in supramolecular structures. Plasma membrane protein extraction using detergents, whilst simultaneously keeping them folded and stable in solution, generates a large set of unknown variables responsible for most of the failures.

Here we have faced this problem for the expression and isolation of integral AQP4-OAPs for structural and functional studies as well as to lay the foundations for future applications for the diagnosis of diseases such as NMO.

To this aim, the choice of the AQP4-isoform (M1- or M23-AQP4) is the crucial point to design a correct expression strategy. Although the use of an expression vector with M1-coding DNA sequence (CDS) would produce variable amount of M23 through leaky scanning and reinitiation mechanisms [17,41] a strategy based on M1 would have provided a poor amount of M23-AQP4 protein and organized in small OAPs [21,42]. Consequently, we have designed a M23-CDS based strategy to maximize the possibility to obtain large, stable, and well AQP4-IgG-recognized OAPs.

Data here reported suggest that the detergent is just one of the fundamental variables. Despite having used the DDM here, the most successful detergent for alpha helical containing membrane protein isolation for crystallography studies [43], we show that the choice of the host-cell type is a pivotal point to isolate integral OAPs.

Here we have explored the possibility of using the DDM/SDS solubility assay to measure the DDM-solubility of M23-AQP4 in OAPs. This assay was previously used to determine the amount of correctly folded G-protein coupled receptor (GPCR), a monomeric membrane protein, in heterologous systems, including Sf9 cells [34]. Here we report that Sf9 cells express a large amount of unfolded AQP4 (DDM-insoluble fraction) and more interestingly that the DDM-solubilised fraction does not contain integral OAPs. This supports the idea that DDM solubility may not be applicable to estimate OAP integrity and suggests that this conclusion may be extended to other plasma membrane proteins characterized by a supramolecular assembly state in the plasma membrane.

The strategy here developed, based on OAP-forming AQP4-M23 isoform expression, allowed us to express OAPs in several cell lines, but the detergent used to extract OAPs revealed crucial differences between these cell lines. In particular, among mammalian cell lines here we showed that the post-extraction OAP-integrity was higher in the adherent flat astrocyte-derived cell line (DI TNC1) compared to HEK-FS. These data suggest a potential role of astrocyte-specific factor or adhesion- or cell-shape-dependent factor in OAP stability. In this respect the cell-shape-dependent actin organization was showed to be determinant for AQP4 localization and, in addition, AQP4 silencing strongly induces cell-shape rearrangement in astrocyte primary cultures [44,45]. Furthermore, deletion of AQP4 C-terminal postsynaptic density 95/disc-large/zona occludens (PDZ) binding domain increased AQP4-M23 diffusion into the cell membrane [46]. These data suggest that actin cytoskeleton or membrane anchoring factors may play a pivotal role in AQP4 localization in the plasma membrane. The role of membrane protein anchoring factors in the OAP assembly is strictly correlated to AQP4 isoform expression. In the natural context the expression pattern of the *AQP4* gene in astrocytes includes at least three other isoforms, M1-AQP4 and two C-terminal extended isoforms named M1ex-AQP4 and M23ex-AQP4, generated by finely regulated alternative transcription/promoters [47] and translational control mechanisms [17,41]. The analysis of OAPs at the level of perivascular astrocyte processes has shown that AQP4 is co-expressed and co-localized with C-terminal extended isoforms. Notably, M23ex-AQP4 is able to physically interact with the canonical AQP4 isoform and with α-syntrophin [6]. α-Syntrophin is a member of the dystrophin-associated protein complex and is required for localization of the OAPs in the perivascular domain at the blood-brain barrier level [48]. It was proposed that the interaction between α-syntrophin and M23ex-AQP4, probably in association with the canonical AQP4, constitutes the dystrophin-dependent perivascular pool of AQP4 [6,49]. BN/SDS-PAGE analysis have largely demonstrated that OAPs contain AQP4ex, and more interestingly for the OAPs post-extraction integrity, that AQP4ex containing OAPs are stable when extracted with DDM starting from CNS tissues [6,37]. These findings suggest that AQP4ex isoforms and/or α-syntrophin may play a pivotal role in the post-extraction stability of OAPs. This suggests that the co-expression of M23-AQP4 with M23ex-AQP4 and/or with human α-syntrophin in Sf9 and HEK-FS may be a strategy to improve the yield of integral post extraction OAPs in these two cell lines.

Our data also demonstrate that the expression of M23-AQP4 in mammalian cells followed by nSEC enrichment is a conservative strategy to obtain integral OAPs and represents a step forward compared to the HIS-tagged based M23-AQP4 purification, which affects OAP stability [23].

Here we have tested the recombinant OAP-integrity by NMO-ELISA. In doing so, we have observed that OAP enriched membranes obtained from M23-AQP4 expressing mammalian cells allow the easy development of a high sensitivity and selectivity ELISA test. Further analysis will be needed to clarify whether this ELISA shows higher performances compared to those that can be obtained by commercial ELISAs and CBA kits already available and largely used into the clinical practice.

Here we found that the AQP4-IgG affinity for AQP4 extracted from M23-AQP4 expressing insect cells was low. These data appear to be in contrast with those obtained with NMO-ELISA based on HIS-tagged AQP4 expressed and purified from insect cells [50,51]. However, it is important to note that in the present study we have measured the affinity of AQP4-IgG binding (AQP4-IgG/AQP4(%)), as indicator of OAP-integrity, and not the absolute binding as reported by other authors. In our hands, by increasing the amount of AQP4/well obtained by M23-AQP4 Sf9 cells we were able to detect AQP4-IgG binding but the ratio between AQP4-IgG binding and AQP4 quantity in the well indicated that only 10% of AQP4 was recognized by AQP4-IgG when the total membrane extract was used (Figure 5c), while this percentage reached the 25% with the nSEC enriched AQP4 from insect cells (Figure 5a). Considering that the M23-AQP4 Sf9 extract does not contain OAPs (Figure 3) and that the AQP4 nSEC-enriched fraction elutes at low molecular weight (Figure 4a), we believe that the low AQP4-IgG affinity measured may be compatible with the less abundant AQP4-IgG population able to recognize OAP-independent linear and intracellular epitopes [33,52,53].

AQP4 expressed and nickel-purified from Sf9 cells has previously been demonstrated to be able to transport water [54]. Considering that water transport is an OAPs-independent function, we believe that the Sf9 expression system may be useful to investigate monomeric- or tetrameric-dependent properties, while mammalian cells and nSEC enrichment are needed to preserve OAP-properties.

Taken together, the data here presented show that the expression of M23-AQP4 in mammalian cells and OAP enrichment by nSEC are mandatory to obtain integral human AQP4-OAPs for downstream applications such as structural and functional studies, crystallography, molecular docking, small molecule inhibitor screening, domain binding property studies, and OAPs-based NMO diagnostic applications. More generally, the strategy here developed may be useful for the expression and isolation of other membrane proteins characterized by a supramolecular assembly state whose molecular architecture and dynamics are still enigmatic.

## References

[1] Abbott N.J., Pizzo M.E., Preston J.E., Janigro D., Thorne R.G. (2018). The role of brain barriers in fluid movement in the CNS: Is there a ‘glymphatic’ system?. Acta Neuropathol..

[2] Nicchia G.P., Nico B., Camassa L.M., Mola M.G., Loh N., Dermietzel R., Spray D.C., Svelto M., Frigeri A. (2004). The role of aquaporin-4 in the blood–brain barrier development and integrity: Studies in animal and cell culture models. Neuroscience.

[3] Nico B., Frigeri A., Nicchia G.P., Quondamatteo F., Herken R., Errede M., Ribatti D., Svelto M., Roncali L. (2001). Role of aquaporin-4 water channel in the development and integrity of the blood–brain barrier. J. Cell Sci..

[4] Frigeri A., Gropper M.A., Turck C.W., Verkman A.S. (1995). Immunolocalization of the mercurial-insensitive water channel and glycerol intrinsic protein in epithelial cell plasma membranes. Proc. Natl. Acad. Sci. USA.

[5] De Bellis M., Pisani F., Mola M.G., Basco D., Catalano F., Nicchia G.P., Svelto M., Frigeri A. (2014). A novel human aquaporin-4 splice variant exhibits a dominant-negative activity: A new mechanism to regulate water permeability. Mol. Biol. Cell.

[6] De Bellis M., Pisani F., Mola M.G., Rosito S., Simone L., Buccoliero C., Trojano M., Nicchia G.P., Svelto M., Frigeri A. (2017). Translational readthrough generates new astrocyte AQP4 isoforms that modulate supramolecular clustering, glial endfeet localization, and water transport. Glia.

[7] Rossi A., Crane J.M., Verkman A.S. (2011). Aquaporin-4 Mz isoform: Brain expression, supramolecular assembly and neuromyelitis optica antibody binding. Glia.

[8] Wolburg H., Berg-von der Emde K., Naujoks-Manteuffel C. (1992). Muller (glial) cells in the retina of urodeles and anurans reveal different morphology by means of freeze-fracturing. Neurosci. Lett..

[9] Wolburg H. (1995). Orthogonal arrays of intramembranous particles: A review with special reference to astrocytes. J. Hirnforsch..

[10] Rossi A., Moritz T.J., Ratelade J., Verkman A.S. (2012). Super-resolution imaging of aquaporin-4 orthogonal arrays of particles in cell membranes. J. Cell Sci..

[11] Smith A.J., Jin B.J., Ratelade J., Verkman A.S. (2014). Aggregation state determines the localization and function of M1- and M23-aquaporin-4 in astrocytes. J. Cell Biol..

[12] Crane J.M., Tajima M., Verkman A.S. (2010). Live-cell imaging of aquaporin-4 diffusion and interactions in orthogonal arrays of particles. Neuroscience.

[13] Verkman A.S., Rossi A., Crane J.M. (2012). Live-cell imaging of aquaporin-4 supramolecular assembly and diffusion. Methods Enzymol..

[14] Verbavatz J.M., Ma T., Gobin R., Verkman A.S. (1997). Absence of orthogonal arrays in kidney, brain and muscle from transgenic knockout mice lacking water channel aquaporin-4. J. Cell Sci..

[15] Frigeri A., Gropper M.A., Umenishi F., Kawashima M., Brown D., Verkman A.S. (1995). Localization of MIWC and GLIP water channel homologs in neuromuscular, epithelial and glandular tissues. J. Cell Sci..

[16] Nicchia G.P., Mastrototaro M., Rossi A., Pisani F., Tortorella C., Ruggieri M., Lia A., Trojano M., Frigeri A., Svelto M. (2009). Aquaporin-4 orthogonal arrays of particles are the target for neuromyelitis optica autoantibodies. Glia.

[17] Rossi A., Pisani F., Nicchia G.P., Svelto M., Frigeri A. (2010). Evidences for a leaky scanning mechanism for the synthesis of the shorter M23 protein isoform of aquaporin-4: Implication in orthogonal array formation and neuromyelitis optica antibody interaction. J. Biol. Chem..

[18] Pisani F., Mastrototaro M., Rossi A., Nicchia G.P., Tortorella C., Ruggieri M., Trojano M., Frigeri A., Svelto M. (2011). Identification of two major conformational aquaporin-4 epitopes for neuromyelitis optica autoantibody binding. J. Biol. Chem..

[19] Pisani F., Mola M.G., Simone L., Rosito S., Alberga D., Mangiatordi G.F., Lattanzi G., Nicolotti O., Frigeri A., Svelto M. (2014). Identification of a point mutation impairing the binding between aquaporin-4 and neuromyelitis optica autoantibodies. J. Biol. Chem..

[20] Crane J.M., Verkman A.S. (2009). Determinants of aquaporin-4 assembly in orthogonal arrays revealed by live-cell single-molecule fluorescence imaging. J. Cell Sci..

[21] Jin B.J., Rossi A., Verkman A.S. (2011). Model of aquaporin-4 supramolecular assembly in orthogonal arrays based on heterotetrameric association of M1-M23 isoforms. Biophys. J..

[22] Rossi A., Baumgart F., van Hoek A.N., Verkman A.S. (2012). Post-Golgi supramolecular assembly of aquaporin-4 in orthogonal arrays. Traffic.

[23] Pisani F., Simone L., Gargano C.D., De Bellis M., Cibelli A., Mola M.G., Catacchio G., Frigeri A., Svelto M., Nicchia G.P. (2017). Role of the H-bond between L53 and T56 for Aquaporin-4 epitope in Neuromyelitis Optica. Biochim. Biophys. Acta.

[24] Rosito S., Nicchia G.P., Palazzo C., Lia A., Buccoliero C., Pisani F., Svelto M., Trojano M., Frigeri A. (2018). Supramolecular aggregation of aquaporin-4 is different in muscle and brain: Correlation with tissue susceptibility in neuromyelitis optica. J. Cell. Mol. Med..

[25] Assentoft M., Kaptan S., Fenton R.A., Hua S.Z., de Groot B.L., MacAulay N. (2013). Phosphorylation of rat aquaporin-4 at Ser(111) is not required for channel gating. Glia.

[26] Ho J.D., Yeh R., Sandstrom A., Chorny I., Harries W.E., Robbins R.A., Miercke L.J., Stroud R.M. (2009). Crystal structure of human aquaporin 4 at 1.8 A and its mechanism of conductance. Proc. Natl. Acad. Sci. USA.

[27] Alberga D., Nicolotti O., Lattanzi G., Nicchia G.P., Frigeri A., Pisani F., Benfenati V., Mangiatordi G.F. (2014). A new gating site in human aquaporin-4: Insights from molecular dynamics simulations. Biochim. Biophys. Acta.

[28] Pisani F., Sparaneo A., Tortorella C., Ruggieri M., Trojano M., Mola M.G., Nicchia G.P., Frigeri A., Svelto M. (2013). Aquaporin-4 autoantibodies in Neuromyelitis Optica: AQP4 isoform-dependent sensitivity and specificity. PLoS ONE.

[29] Kitley J., Woodhall M., Leite M.I., Palace J., Vincent A., Waters P. (2015). Aquaporin-4 antibody isoform binding specificities do not explain clinical variations in NMO. Neurol. Neuroimmunol. Neuroinflamm..

[30] Tuller F., Holzer H., Schanda K., Aboulenein-Djamshidian F., Hoftberger R., Khalil M., Seifert-Held T., Leutmezer F., Berger T., Reindl M. (2016). Characterization of the binding pattern of human aquaporin-4 autoantibodies in patients with neuromyelitis optica spectrum disorders. J. Neuroinflamm..

[31] Majed M., Fryer J.P., McKeon A., Lennon V.A., Pittock S.J. (2016). Clinical utility of testing AQP4-IgG in CSF: Guidance for physicians. Neurol. Neuroimmunol. Neuroinflamm..

[32] Jarius S., Paul F., Fechner K., Ruprecht K., Kleiter I., Franciotta D., Ringelstein M., Pache F., Aktas O., Wildemann B. (2014). Aquaporin-4 antibody testing: Direct comparison of M1-AQP4-DNA-transfected cells with leaky scanning versus M23-AQP4-DNA-transfected cells as antigenic substrate. J. Neuroinflamm..

[33] Jarius S., Wildemann B. (2013). Aquaporin-4 antibodies (NMO-IgG) as a serological marker of neuromyelitis optica: A critical review of the literature. Brain Pathol..

[34] Thomas J.A., Tate C.G. (2014). Quality control in eukaryotic membrane protein overproduction. J. Mol. Biol..

[35] Wingerchuk D.M., Lennon V.A., Pittock S.J., Lucchinetti C.F., Weinshenker B.G. (2006). Revised diagnostic criteria for neuromyelitis optica. Neurology.

[36] Polman C.H., Reingold S.C., Banwell B., Clanet M., Cohen J.A., Filippi M., Fujihara K., Havrdova E., Hutchinson M., Kappos L. (2011). Diagnostic criteria for multiple sclerosis: 2010 revisions to the McDonald criteria. Ann. Neurol..

[37] Nicchia G.P., Rossi A., Mola M.G., Pisani F., Stigliano C., Basco D., Mastrototaro M., Svelto M., Frigeri A. (2010). Higher order structure of aquaporin-4. Neuroscience.

[38] Schagger H., Cramer W.A., von Jagow G. (1994). Analysis of molecular masses and oligomeric states of protein complexes by blue native electrophoresis and isolation of membrane protein complexes by two-dimensional native electrophoresis. Anal. Biochem..

[39] Mola M.G., Nicchia G.P., Svelto M., Spray D.C., Frigeri A. (2009). Automated cell-based assay for screening of aquaporin inhibitors. Anal. Chem..

[40] Pisani F., Settanni P., Rosito S., Mola M.G., Iorio R., Tortorella C., Ruggieri M., Trojano M., Svelto M., Frigeri A. (2015). Development of an Aquaporin-4 Orthogonal Array of Particle-Based ELISA for Neuromyelitis Optica Autoantibodies Detection. PLoS ONE.

[41] Pisani F., Rossi A., Nicchia G.P., Svelto M., Frigeri A. (2011). Translational regulation mechanisms of aquaporin-4 supramolecular organization in astrocytes. Glia.

[42] Furman C.S., Gorelick-Feldman D.A., Davidson K.G., Yasumura T., Neely J.D., Agre P., Rash J.E. (2003). Aquaporin-4 square array assembly: Opposing actions of M1 and M23 isoforms. Proc. Natl. Acad. Sci. USA.

[43] Parker J.L., Newstead S. (2016). Membrane Protein Crystallisation: Current Trends and Future Perspectives. Adv. Exp. Med. Biol..

[44] Nicchia G.P., Rossi A., Mola M.G., Procino G., Frigeri A., Svelto M. (2008). Actin cytoskeleton remodeling governs aquaporin-4 localization in astrocytes. Glia.

[45] Nicchia G.P., Frigeri A., Liuzzi G.M., Svelto M. (2003). Inhibition of aquaporin-4 expression in astrocytes by RNAi determines alteration in cell morphology, growth, and water transport and induces changes in ischemia-related genes. FASEB J..

[46] Crane J.M., Van Hoek A.N., Skach W.R., Verkman A.S. (2008). Aquaporin-4 dynamics in orthogonal arrays in live cells visualized by quantum dot single particle tracking. Mol. Biol. Cell.

[47] Abe Y., Goda W., Ikeshima-Kataoka H., Yasui M. (2017). The dual effects of the astrocyte-specific enhancer of the AQP4 M1 promoter. FEBS Lett..

[48] Neely J.D., Amiry-Moghaddam M., Ottersen O.P., Froehner S.C., Agre P., Adams M.E. (2001). Syntrophin-dependent expression and localization of Aquaporin-4 water channel protein. Proc. Natl. Acad. Sci. USA.

[49] Nicchia G.P., Rossi A., Nudel U., Svelto M., Frigeri A. (2008). Dystrophin-dependent and -independent AQP4 pools are expressed in the mouse brain. Glia.

[50] Hayakawa S., Mori M., Okuta A., Kamegawa A., Fujiyoshi Y., Yoshiyama Y., Mitsuoka K., Ishibashi K., Sasaki S., Hattori T. (2008). Neuromyelitis optica and anti-aquaporin-4 antibodies measured by an enzyme-linked immunosorbent assay. J. Neuroimmunol..

[51] Kim Y.J., Jung S.W., Kim Y., Park Y.J., Han K., Oh E.J. (2012). Detection of anti-aquaporin-4 antibodies in neuromyelitis optica: Comparison of tissue-based and cell-based indirect immunofluorescence assays and ELISA. J. Clin. Lab. Anal..

[52] Yu X., Green M., Gilden D., Lam C., Bautista K., Bennett J.L. (2011). Identification of peptide targets in neuromyelitis optica. J. Neuroimmunol..

[53] Kampylafka E.I., Routsias J.G., Alexopoulos H., Dalakas M.C., Moutsopoulos H.M., Tzioufas A.G. (2011). Fine specificity of antibodies against AQP4: Epitope mapping reveals intracellular epitopes. J. Autoimmun..

[54] Yang B., van Hoek A.N., Verkman A.S. (1997). Very high single channel water permeability of aquaporin-4 in baculovirus-infected insect cells and liposomes reconstituted with purified aquaporin-4. Biochemistry.

